# How do patient characteristics influence informal payments for inpatient and outpatient health care in Albania: Results of logit and OLS models using Albanian LSMS 2005

**DOI:** 10.1186/1471-2458-11-375

**Published:** 2011-05-23

**Authors:** Sonila Tomini, Hans Maarse

**Affiliations:** 1 Maastricht Graduate School of Governance, Maastricht University, Maastricht, the Netherlands; 2 Department of Health Organization Policy and Economics, Faculty of Health Medicine and Life Sciences, Maastricht University, Maastricht, the Netherlands

**Keywords:** Public health care, informal payments, economic transition, governance, Albania

## Abstract

**Background:**

Informal payments for health care are common in most former communist countries. This paper explores the demand side of these payments in Albania. By using data from the Living Standard Measurement Survey 2005 we control for individual determinants of informal payments in inpatient and outpatient health care. We use these results to explain the main factors contributing to the occurrence and extent of informal payments in Albania.

**Methods:**

Using multivariate methods (logit and OLS) we test three models to explain informal payments: the cultural, economic and governance model. The results of logit models are presented here as odds ratios (OR) and results from OLS models as regression coefficients (RC).

**Results:**

Our findings suggest differences in determinants of informal payments in inpatient and outpatient care. Generally our results show that informal payments are dependent on certain characteristics of patients, including age, area of residence, education, health status and health insurance. However, they are less dependent on income, suggesting homogeneity of payments across income categories.

**Conclusions:**

We have found more evidence for the validity of governance and economic models than for the cultural model.

## Background

Informal payments to health service providers are widespread not only in the former communist countries (Central and Eastern Europe and Former Soviet Union), but also in other middle income and developing countries [1-6]. These unregulated out-of-pocket payments are often labelled as 'under the table payments', 'envelope payments', 'under-the-counter payments', 'gifts', and even 'black money'. They are usually defined as payments made to health care providers for services which are supposed to be provided at no charge to the patient [4] and may take different forms, from cash payments to in-kind contributions and from gift giving to informal charging [6].

Informal payments in health care can be conceptualised as a strategy to cope with lack of resources and poor performance at both the demand and supply side [4,7]. Patients may pay informally to jump the queue, to receive higher quality of services or more care [4,7,8]. Providers may use informal payments to raise their low salaries.

Informal payments were very common in Albania in 2005, especially in inpatient health care, which because of state funding was officially free of charge for the entire population. The situation was particularly dramatic for people in the lowest quintile of income distribution [9].

This paper examines how patients' characteristics influence informal payments for outpatient and inpatient care. We test three models (*i*) the cultural model, (*ii*) the economic model and (*iii*) the governance model to explain the occurrence and extent of informal payments in Albania. In our analysis we make use of the Albania Living Standards Measurement Survey (ALSMS) of 2005. Our intention is to explore who pays, for what, how much, and when is the payment voluntary or requested/expected.

## Methods

The causes and origins of informal payments have been a topic of frequent discussion. Various factors have been suggested to explain informal payments including: a) the culture of gifts [7,10,11]; b) the low salary of medical staff [12-16]; c) the lack of resources and material supplies [10,13,14]; d) the bargaining power of medical staff relative to patients [17,18]; and e) the lack of regulation and unresponsiveness of the government [13,15]. We base our theoretical framework on the main explanatory factors for informal payments identified by Gaal and McKee [19]. We group them in three models: (*i*) the cultural model, (*ii*) the economic model, and (*iii*) the governance model (see also Table 1).

**Table 1:** Causes of informal payments and observed effects

Models	Causal factor	Observed effects
1. Cultural	1. Culture of gratitude gifts and tipping	1. Money and in kind gifts paid to medical staff (usually after the services received). 2. Patients declare gifts to medical staff mostly as voluntary. 3. A relatively moderate incidence of such payments, usually of modest value.
2. Economic	1. Demand side factors -free heath care access 2. Supply side factors -Inadequate funding -Low salaries of health care staff. -Investments oriented more towards access rather than quality of services.	1. Unequal amounts paid for inpatient and outpatient health services. 2. Informal payments resemble a 'fee-for-service' model (as patients may use informal payments as top ups or for getting better quality of service). 3. High income patients will pay higher informal payment. 4. Having health insurance will lower the probability of paying informally.
3. Governance	1. Lack of control and accountability leading to unethical behaviour of medical staff. 2. Weak rule of law and corruption control.	1. High incidence of explicitly requested payments. 2. Probability of paying informally is dependent on the knowledge and bargaining power of the patients. 3. Low numbers of prosecuted cases of corrupted medical staff.

The cultural model considers informal payments a particular type of behaviour where care seekers express their gratitude in the form of gifts. Such behaviour is found in many countries, in particular in Mediterranean, CEU and FSU countries. The 'culture of gifts' roots in values and traditions [11] and is conceptualised as institutionalised behaviour of patients. Seen as voluntary behaviour, it is sometimes argued that it does not put any particular burden on them. The value of gifts is supposed to be modest and directly linked to their ability to pay. Payments are expected to improve the motivation of the medical staff, ensure a personal relation, and provide incentives for physicians to feel appreciated [5].

A good strategy to measure the culture of gift is to ask patients about their attitude to gift payments in health care and the amount they would consider appropriate. Unfortunately, the ALSMS 2005 does not contain data on the patients' attitude. Therefore, we used proxy indicators. Typical for the culture of gift is that informal payments are widespread and perceived as voluntary. Furthermore, we expect the amount paid to be relatively low compared to the total amount paid out-of-pocket. There is also a positive correlation between the patient's income and the amount paid.

The economic model conceptualises informal payments as the result of a gap between demand and supply (lack of financial resources). The communist regimes established in CEE and FSU countries after the Second World War propagated free access to health care policy under the legacy of the 'Shemasko' health care system. Free access boosted the demand for health care which in turn caused a shortage of supply [19]. After the fall of communist regimes, governments in these countries were not capable to maintain an inefficiently operating health care system that guaranteed free access to all patients. With state revenues declining in many CEE and FSU countries in the early 1990s, health expenditures also dramatically fell. Due to political circumstances the number of staff and beds remained largely unchanged [13,15]. The inevitable result was a large health system with underpaid and sometimes even unpaid staff. Albania's limited public spending on health care sector has resulted in an increased reliance on out-of-pocket payments for both inpatient and outpatient care [20]. According to the WHO, in 2005 out-of-pocket expenditures accounted for 59.8 percent of Albania's total health expenditures [21]. Furthermore, a rural-to-urban brain drain [22] caused a physician shortage in rural areas and small towns (especially in inpatient care). The mismatch between demand and supply in health care and the unresponsive character of the health system led to a situation in which patients used informal payments to get (quick) access to care and medical staff used informal payments to increase revenues. Due to political circumstances the situation further worsened by the fact that investments continued to be more access-driven than quality-driven [20]. Patients were more willing to pay extra for what they perceived as better quality of care.

In the economic model we expect informal payments to be higher in inpatient care than in outpatient care. This expectation rests upon the assumption that the severity of illness of patients who need outpatient care is on average less than the severity of illness of patients in need of inpatient care. Informal payments are also expected to be higher for hospital doctors than for general practitioners. Informal payments will also positively correlate with income. Patients who are covered by health insurance will be less willing to make informal payments, because they know they have already paid for medical care.

Informal payments may also point to poor governance. The governance model links informal payments to lack of control and accountability in health care, weak rule of law and widespread corruption [19]. Each of these factors elicits non-ethical behaviour of the medical staff. Patients are not protected by public authorities and, as a consequence, are more exposed to exploitation from physicians. In spite of their official commitment to the professional model which states that patient interests should always prevail over the physician's economic interest [23,24], physicians are tempted to abuse the asymmetric physician-patient relationship to increase their earnings [17,18].

If informal payments are the result of poor governance, we expect (*i*) a high incidence of explicitly requested payments (direct payments to physicians are forbidden by law in Albania: therefore, asking explicitly for a gift/payment can be taken as an indicator of unethical behaviour), (*ii*) the probability of informal payments to be related to the knowledge and bargaining power of patients (people with low education will have less negotiating power and may therefore easily be exploited by medical staff), and (*iii*) tolerance of corruption (i.e. number of prosecuted cases for corrupt medical staff is very low).

Our data were taken from the Albania Living Standard Measurement Survey 2005 which is freely accessible on the World Bank's website (http://www.worldbank.org/lsms). ALSMS uses a stratified sample and distinguishes four main areas; Tirana (the capital of the country) and three other agro-ecological/economic areas (Coastal, Central and Mountainous). The survey is representative for Tirana and the other three areas as well as for urban-rural areas [25]. The numbers of households sampled in 2005 is 3640 (17302 individuals). Data are collected both for individuals and households and variables concern demographic, social and economical characteristics. The data set includes detailed information on households' income and expenditures for housing, food consumption, education, and so on.

The set of questions of our interest refers to whether individuals utilised health care in the past period (4 weeks for outpatient care and 12 months for inpatient care). Information on informal payments concerns the total amount paid to the medical staff^1 ^and the voluntary or requested character of these payments. Out of the total sample, 2244 individuals reported to have received outpatient care (1591 in public ambulatory clinics and 653 in hospital outpatient departments) and 710 inpatient care. Data collected by the ALSMS 2005 include all informal payments under "gifts paid to medical staff". Table 2 gives a complete overview of the variables used in our models.

**Table 2:** Descriptive statistics

	The incidence of paying informally				The amount paid informally			
Variables	Outpatients services		Inpatients services		Outpatients services		Inpatients services	
Amount paid informally (in Lek) *	-	-	-	-	658.042	(2473.042)	1298.988	(2499.494)
Household size	4.594	(1.994)	5.028	(1.935)	4.731	(2.100)	5.249	(2.060)
Age of the individual	46.679	(24.573)	40.699	(22.818)	42.882	(25.413)	39.040	(23.211)
Gender (Female = 1)	0.583	(0.493)	0.577	(0.494)	0.617	(0.487)	0.599	(0.491)
Urban/Rural (Rural = 1)	0.450	(0.498)	0.557	(0.497)	0.519	(0.500)	0.590	(0.492)
Income/capita	9160.373	(7277.878)	8016.400	(6121.248)	8644.299	(6552.127)	7900.646	(6211.548)
Health Insurance (Without health insurance = 1)	0.625	(0.484)	0.486	(0.500)	0.540	(0.499)	0.404	(0.491)
Health rate (1 'v. poor' - 5 'v. good')	2.932	(0.983)	3.151	(1.149)	2.993	(0.979)	3.189	(1.152)
Chronic illness (Chronic illness = 1)	0.663	(0.473)	0.472	(0.500)	0.608	(0.489)	0.421	(0.494)
Without education (Without education = 1)	0.011	(0.105)	0.004	(0.067)	0.013	(0.114)	0.003	(0.053)
Education 8-years (Education 8-years = 1)	0.523	(0.500)	0.581	(0.494)	0.554	(0.498)	0.588	(0.493)
Secondary education (Secondary education = 1)	0.200	(0.400)	0.204	(0.403)	0.175	(0.380)	0.186	(0.390)
University education (University education = 1)	0.055	(0.227)	0.045	(0.207)	0.015	(0.123)	0.045	(0.208)
Hospitals in Tirana (Visiting in Tirana hospital = 1)	-	-	0.323	(0.468)	-	-	0.302	(0.460)
Hospital in the same district (Visiting in the same hospital district = 1)	-	-	0.528	(0.500)	-	-	0.599	(0.491)
Hospital in different district (Visiting in the different hospital = 1)	-	-	0.124	(0.329)	-	-	0.096	(0.295)
Hospital in foreign country (Visiting in foreign country hospital = 1)	-	-	0.025	(0.157)	-	-	0.003	(0.053)
Rural/Urban * Hospital in Tirana district (Rural patient visit in Tirana hospital = 1)	-	-	0.128	(0.335)	-	-	0.124	(0.330)
Nr of times visit outpatients care/Days stayed in Hospital	1.393	(0.933)	16.171	(20.154)	1.418	(0.966)	15.477	(17.744)
The incidence of paying informally (Paying informally = 1)	0.233	(0.423)	0.528	(0.500)	-	-	-	-
The incidence of voluntary/requested payments (Requested payments = 1)	-	-	-	-	0.617	(0.487)	0.686	(0.465)
Number of Observations	1963		671		457		354	

Note: *) In inpatient care the amount paid informally is the average per day in hospital.

In outpatient care the amount paid informally is the average per visit.

Standard errors are in bracket

100 ALL = 0.80 Euros in June 2005

In order to investigate the determinants of informal payments in inpatient and outpatient care we estimate a logit model to analyse the probability of payments and an OLS model to analyse the amount paid. In our analysis we try to distinguish between gratuities and informal payments by controlling for their main drivers and estimating a separate model controlling for the voluntary/requested character of them. Our models are estimated separately for inpatient and outpatient care (see also Table 3). Inpatient care includes all visits in public hospitals over the past 12 months and outpatient care all visits in public ambulatories and outpatient departments of public hospitals during the past 4 weeks^2^. Informal payments for inpatient care are measured as the average amount paid per day and informal payments in outpatient care as the average amount paid per visit.

**Table 3:** Logit and OLS estimations of the probability and the amount paid as informal payments to medical staff

	Odds ratio of paying informally to Medical Staff				The amount of informal payments paying to Medical Staff				Odds ratio if informal payments have been voluntary/requested by Medical Staff			
	Outpatient service		Inpatient service		Outpatient service		Inpatient service		Outpatient Service		Inpatient service	
Household size	0.996	(0.030)	1.112**	(0.050)	0.024	(0.022)	-0.071**	(0.035)	0.870***	(0.047)	0.939	(0.061)
Age of the individual	0.994**	(0.003)	0.999	(0.005)	0.002	(0.002)	0.003	(0.004)	0.994	(0.006)	0.989	(0.007)
Gender	1.166	(0.131)	1.136	(0.190)	0.043	(0.087)	0.019	(0.144)	0.598**	(0.128)	0.771	(0.203)
Urban/Rural	1.226*	(0.147)	0.968	(0.215)	0.202**	(0.091)	0.017	(0.192)	1.375	(0.303)	1.350	(0.465)
Log income/capita	1.046	(0.075)	1.080	(0.112)	0.075	(0.057)	0.110	(0.087)	0.733**	(0.107)	0.914	(0.151)
Health insurance	0.782**	(0.094)	0.542***	(0.101)	-0.375***	(0.091)	-0.071	(0.164)	0.998	(0.224)	0.962	(0.293)
Health rate (1 'v. poor' - 5 'v. good')	0.993	(0.069)	0.827*	(0.089)	-0.033	(0.058)	0.292***	(0.094)	0.898	(0.126)	0.789	(0.139)
Chronic illness	0.980	(0.155)	0.654*	(0.157)	-0.030	(0.123)	0.388*	(0.217)	1.733*	(0.515)	1.291	(0.515)
Education primary	1.124	(0.166)	1.074	(0.229)	-0.091	(0.114)	-0.092	(0.184)	1.417	(0.382)	1.839*	(0.597)
Education university	0.292***	(0.120)	1.332	(0.558)	0.195	(0.353)	1.026***	(0.357)	2.783	(2.502)	1.167	(0.748)
Hospital in Tirana district			0.943	(0.228)			0.253	(0.214)			0.706	(0.263)
Rural/Urban x Hospital in Tirana district			1.044	(0.370)			0.231	(0.313)			2.785*	(1.714)
Nr. of times visit outpatient health service/Days stayed in hospital	1.064	(0.061)	0.998	(0.004)	-0.037	(0.044)	-0.033***	(0.004)	0.892	(0.094)	1.006	(0.009)
Log informal amount paid					-	-	-	-	1.197	(0.140)	1.663***	(0.177)
Constant					5.184***	(0.715)	4.627***	(1.134)				
												
Number of observations (N)	1963		671		457		354		457		354	
R-squared					0.2191		0.3088					
Pseudo R2	0.0257		0.036						0.0001		0.0001	

Note: .01 - ***; .05 - **; .1 - *. Standard errors are in bracket

To understand informal payments better, we further investigate the relationship between informal payments for inpatient and outpatient care. Are persons who pay for one type of service more likely to pay for the other type as well? Are the amount paid for inpatient and outpatient care correlated? To answer these questions, we investigate the correlation of the residuals of our logit and OLS models (see Table 4).

**Table 4:** Correlation of residuals from the probability and amount models

	Logit of paying informally in Inpatient service	Amount paid informally in Inpatient service
Logit of paying informally in Outpatient services	0.648** (247)	n.a.
Amount paid informally in Outpatient service	n.a.	''0.006 (60)

Note: .01 - ***; .05 - **; .1 - *. Numbers of observations are in bracket

## Results

The results from logit and OLS models are given in Table 3. Our findings indicate that the incidences and the amounts paid informally to medical staff vary across patient categories.

Elderly patients seeking outpatient health care had a slightly lower probability of paying informal payments (OR = 0.99; p-value = 0.048). The demand for health care rises with age and therefore the elderly were more likely to need health care. They were also more likely to have health insurance (e.g. universal coverage for pensioners) and to belong to lower income quintiles (World Bank, 2006). In our model, both factors lowered the probability of informal payments for outpatient care. The probability of informal payments for inpatient care was also slightly lower, but not statistically significant (OR = 0.99; p-value = 0.750).

Patients living in rural areas and visiting outpatient care seemed more likely to make informal payments than patients living in urban areas (OR = 1.22; p-value = 0.090). They also paid more informally than patients living in urban areas (RC = 0.121; p-value = 0.027). In 2005, many of the health care centres in rural areas were non-functional or did not even offer basic services [9]. As a consequence, most people living in these areas preferred the facilities in big urban centres, where, due to absence of a referral or social connections, the pressure to pay was higher [16]. A slightly different situation was observed for inpatient care (though results were not statistically significant). Here, those living in rural areas seemed less likely to pay (OR = 0.96; p-value = 0.884), but more likely to pay higher amounts (RC = 0.017; p-value = 0.928) than patients living in urban areas. For these patients the treatment was associated with additional opportunity costs (travelling, accommodation expenses for the relatives accompanying, etc) and the medical staff seemed to take more advantage of such aspects.

The not statistically significant role of income for explaining the probability of informal payments in outpatient and inpatient care (OR = 1.05; p-value = 0.524; OR = 1.08; p-value = 0.459) and the amount paid informally (RC = 0.075; p-value = 0.195; RC = 0.110; p-value = 0.207) indicates that all patients were likely to be affected by informal payments regardless of their income.

**Figure 1:**
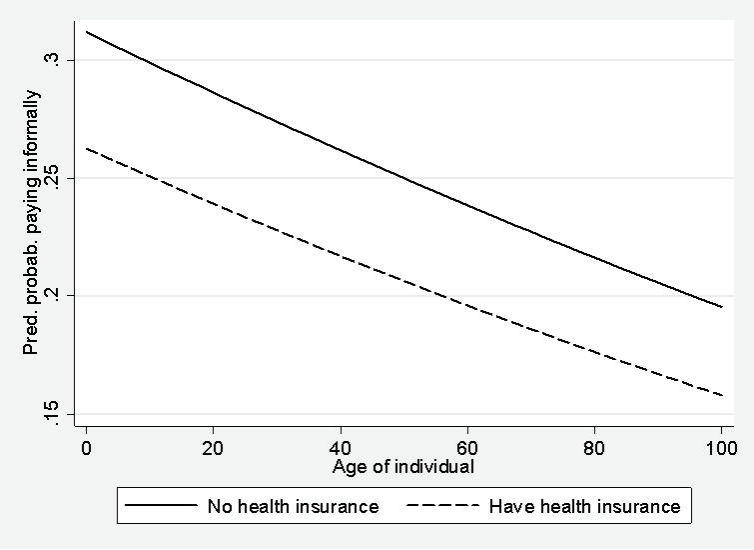
**Outpatient care - predicted probability of paying informally by age group with and without health insurance**.

**Figure 2:**
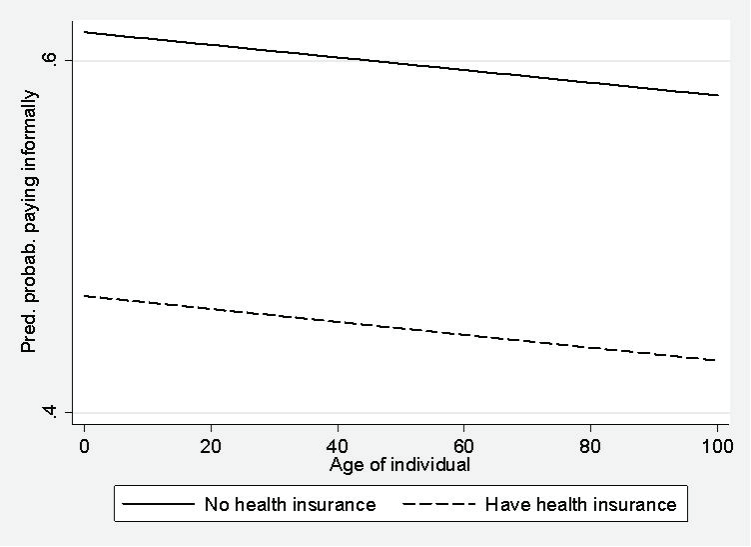
**Inpatient care - predicted probability of paying informally by age group with and without health insurance**.

Patients with health insurance were less likely to pay informally for inpatient (OR = 0.78; p-value = 0.041) and outpatient care (OR = 0.54; p-value = 0.001) than uninsured patients. This was also due to their greater bargaining power in interacting with the medical staff. Such patients knew the rules of the game in hospitals and were better informed on what is covered. Figure 1 and Figure 2 show the probability of paying informally in outpatient and inpatient care for all age groups with or without health insurance. Younger contributors were more likely to pay, while health insurance reduced the probability of informal payments across all ages. However, we believe that the particular form of service contracting and the financing in outpatient services shaped the curves differently. Older patients were less likely to pay informally for outpatient care than for inpatient care. Health insurance reduced this probability in outpatient care as this care was mostly funded by Health Insurance Institute. The probability of informal payments remained high for inpatient care.

Patients with lower score on self-reported health were more likely to make informal payments in inpatient care than other patients (OR = 0.83; p-value = 0.074). The same also holds for patients with chronic illnesses compared to patients with acute diseases (OR = 0.65; p-value = 0.077). However, both groups seemed to pay lower amounts for inpatient care.

Patients with chronic illness were more likely requested to make an informal payment for outpatient care than all other patients (OR = 1.73; p-value = 0.065) and lower educated people were more likely requested to make an informal payment for inpatient care (OR = 1.84; p-value = 0.061) than people with secondary or university education. A similar result was found for rural residents treated in Tirana's hospitals. Rural patients seeking inpatient care in Tirana (the capital of the country and the only city offering tertiary care) were also more likely requested/expected explicitly by the medical staff to make an informal payment than urban patients (OR = 2.79; p-value = 0.096).

People with university education were less likely to pay in outpatient care (OR = 0.29; p-value = 0.003), but paid a higher amount for inpatient care (RC = 1.025; p-value = 0.004). These categories were more likely to have health insurance (because of their formal employment) and also earned higher income. They were better covered in outpatient care, where the role of health insurance was stronger, but their higher informal payments in inpatient services suggested that they were purchasing upgrades and/or more attention. When testing whether these payments were voluntarily or requested, we found that patients with primary education were more frequently requested to pay informally.

When testing for the location of the hospital, the results suggested that informal payments were more likely higher in Tirana's hospitals (RC = 1.026; p-value = 0.238). This result is not very surprising. Hospitals in Tirana employ the elite of the medical staff and offer specialised treatments.^3 ^An additional explanation may be the absence of social relations between patients and physicians in a big city as Tirana.

The numbers of days in hospital had a significant and negative influence on the amount paid informally (RC=-0.033; p-value = 0.000). High utilization patients seemed to exhaust their resources and/or became more familiar with the informal system of paying. Moreover, estimates showed that the higher the amount of informal payments, the more likely it was to be 'explicitly requested' (OR = 1.66; p-value = 0.000).

Patients paying for outpatient care were also likely to pay for inpatient care (see Table 4). This suggests that certain characteristics of patients highly influenced the probability of payment for these services (correlation coefficient = 0.648; p-value = 0.000).^4 ^However, informal payments paid for inpatient care were not statistically correlated to the amount paid for outpatient care (correlation coefficient = 0.006; p-value = 0.966). Our survey shows that the average amount paid informally for inpatient care was several times higher than the amount paid informally for outpatient care (see also Table 2).

## Discussion

The results from the logit and OLS models are summarised in Table 5. The table shows which of the three models (cultural, economic and governance) offers the best explanation for informal payments.

**Table 5:** Main empirical findings and the evidence on the three models

Findings	The social-cultural model	The economic model	The governance model
1. Informal payments are widespread in outpatient and inpatient services.	+	-	+
2. More patients (52.8%) pay informal payments in inpatient care than in outpatient care (23.3%).	-	+	+/-
3. The amounts paid in inpatient are larger than in outpatient care (fee-for-service)	-	+	-
4. Almost 61.7 of the payments in outpatient and 68.6% in inpatient care are expected or requested.	-	-	+
5. Positive but not statistically significant association of per capita income with probability and amounts of informal payments.	-	+/-	+/-
6. The elderly are less likely to make informal payments (especially in outpatient).	-	+	+/-
7. Rural areas patients seeking care in Tirana are more likely to pay and to have been explicitly requested an informal payment.	-	+/-	+
8. Vulnerable patients (higher ages and poor health rated) are more likely to pay in inpatient care but not in outpatient care.	-	-	+
9. Higher amount paid in hospitals located in Tirana.	-	+	-
10. Rural patients visiting Tirana's hospitals are more likely to be requested to make informal payments.	-	+/-	+
11. Higher educated patients pay more in inpatient care where the role of health insurance is weak and services are more intense.	-	+	+/-
12. Primary education patients are requested to pay in inpatient services.	-	-	+

Note: The positive and negative signs in the table indicate if the evidence found from the data analysis (logit and OLS models in Table 3) support or not each of the models.

Our empirical findings suggested that informal payments were widespread in both outpatient and inpatient care. Patients paid informally to medical staff either because they felt that they should pay (voluntary) or because they were obliged to do so (requested/expected). This clearly supports both cultural and governance models. However, we also found that determinants of informal payments for inpatient and outpatient care appeared to be different. These differences were not only associated with the type of health care, but also with the extent of coverage by health insurance. The Health Insurance Institute had contractual agreements covering only outpatient care, while inpatient care was state-funded. ^5 ^Patients were more likely to pay for inpatient than for outpatient care (52.8% of them declared to have paid for inpatient care compared to 23.3% for outpatient care). This result contrasts with the cultural model since this model predicts no differences in the probability of informal payments for inpatient and outpatient care.

The findings also suggested that patients with chronic illnesses, the lower educated, or rural residents treated in Tirana's hospitals were more likely requested to pay informally to medical staff. These findings are not in accordance with the cultural model, but can be interpreted as empirical evidence in favour of the economic and governance models. Because the government fails to protect patients in these categories, they are exposed to the rules of the market (If you need care you have to pay it!). The high level of explicitly requested payments from patients in these categories points to an overall lack of accountability in both outpatient and inpatient care. This result supports the governance model.

The differences we found in the amounts paid informally in outpatient and inpatient care suggest that such amounts may depend on the specific treatment. Moreover, estimates show that the higher the amount of informal payments, the more likely it is to be 'explicitly requested'. These findings also support the economic and governance models which conceptualise informal payments as a fee-for-service and a consequence of medical staff's unethical behaviour as well as an inefficiently operating and poorly governed system.

The elderly patients were less likely to pay in outpatient care than the younger ones. We argued that this may be because they were more likely to have health insurance (e.g. universal coverage for pensioners) and belonged to lower income quintiles (World Bank, 2006). The result contrasts with the cultural model, but is in accordance with the economic and (partly) the governance model (the elderly make more visits but have better insurance coverage and less financial resources).

The weak relation between patient's income and probability of paying informally can be considered to be a governance failure causing inequity in health care (everyone has a certain probability for paying informally regardless of income). However, the positive association of income with the amount paid suggests that high income patients pay more informally for health care. This finding offers some support for the economic model and is in line with other studies on informal payments in Albania [7].

An interesting finding was that patients with lower rated health status were more likely to pay. This could be explained by their income level and societal position. As they belong mostly to lower income categories (health negatively correlates with income), they may have lower bargaining power and hence be more likely to pay informally. However, we should consider that they may have not paid for better service but for getting access [4]. This could explain the lower amount paid. These findings support the governance model according to which medical staff requests informal payments more often from vulnerable patients because of their lower bargaining power.

The high dependence of informal payments on the health insurance status in both outpatient and inpatient care demonstrates again, that the most vulnerable are also more likely to be exploited. Lack of governmental protection and lower negotiating power make them more vulnerable to informal payments. Higher educated patients have more 'information' on how the health system works, how much the medical staff gets, and how much the additional services (or better services) cost. In fact, having higher education may help in getting better negotiating position with medical staff.

Although our study sheds more light on the demand side of informal payments, it has some limitations because of the available data. Albania LSMS 2005 does not allow us to distinguish between payments made to physicians or other staff, nor does it capture the payments made in kind or through favours rendered. Our analysis is based on the information on "gifts made to medical staff". The questionnaire does not ask explicitly for the total amount of informal payments (which would also include other payments, for example payments for pharmaceuticals that otherwise were suppose to be free). Furthermore, we are not able to determine to what extent social connections (i.e. knowing the medical staff beforehand) influence informal payments.

In this study we have mostly concentrated on patient characteristics. The data gives limited information on the behaviour of the medical staff. Information on the hierarchical position of physicians or their particular professional background would enable us to get more insight into who is requesting and who gets what. Moreover, linking information on the culture towards informal payments in particular health centres or hospitals (e.g. number of penalised staff for practicing informal payments) would give a better overview of the rule of law and its effects on the incidence and amounts paid.

## Conclusion

In this paper we tested the cultural, economic and governance models explaining the occurrence and extent of informal payments. Taking into consideration that Albania is a Mediterranean country, we expected the cultural model to be the best model for explaining informal payments. However, our data and results do not confirm this expectation. Our findings provide more evidence for the economic and governance models.

The economic model favours mostly a 'fee-for-service' description of informal payments. They fill the gap due to lack of resources. The unequal amount paid for inpatient and outpatient care, the low correlation of amounts paid informally in both services and the higher amount paid informally in hospitals located in Tirana appear to support the economic model. Better trained physicians (concentrated in Tirana's hospitals) seem to seek more financial rewards than their colleagues. Higher educated patients pay more for inpatient care where the role of health insurance is weak and services are more intense. These findings may suggest that informal payments are meant to purchase better care.

It is difficult to draw a clear-cut diving line between the economic and governance model. To some extent they can be seen as a consequence of each other. Low investments, a low budget for health care and inequalities between rural and urban areas may imply some of the main causes of informal payments. These factors can be understood as motivating the medical staff to request informal payments. Informal payments may also be seen as the result of the lack of accountability and the weak rule of law in Albania. Public authorities are reluctant to take effective measures to punish them for their behaviour. Lack of support to vulnerable groups (i.e. chronically ill or uninsured) and low levels of information further increase the negative effects of informal payments for these particular groups.

Patients and physicians are using informal payments as a coping strategy to ease problems caused by lack of resources and poor governance [15]. We showed that the burden of these payments falls mostly on the most vulnerable groups in society. From a policy perspective, the most effective measure would be to include inpatient health care in the benefit package of the national health insurance scheme. Widening the coverage of health insurance and direct contracting inpatient care would help to increase efficiency of health care provision. The implementation of this change has only started in 2009. Its effects on inpatient care are yet to be seen. However, the extension of health insurance to inpatient care is not sufficient as a separate policy intervention. It must be combined with policy measures to protect the chronically ill and the uninsured. Experience from other countries has shown that the formalisation of informal payments alone has not eradicated corruption in the health sector [26]. It goes without saying that the increased efficiency in financing health care system would also require measures to improve the quality of the services.

## Competing interests

The authors declare that they have no competing interests.

## Authors' contributions

ST designed and conceptualised the study and conducted the data analysis. HM conceptualised the discussion and contributed to the discussion and conclusion sections. All authors approved the final manuscript.

## Endnotes

^1 ^Albania LSMS 2005 gathers specific information on gifts made to medical staff. Such gifts are considered informal in Albania's health care context and are not allowed by the legislation in place. We have used this as the best possible measure of informal payments in the country for year 2005.

^2 ^Due to the low prevalence of informal payments in private doctors and dentist care we do not estimate models for these private services.

^3 ^This has created large inequalities between regions in the access to quality services [22].

^4 ^Based on the results of logit models, we can single out health insurance as the most evident variable (negative and significant for both models): people without insurance were more likely to be exploited in both services.

^5 ^In 2005 the covered categories included those paying contributions, pensioners, children 0-14 years old, students, soldiers, disabled, unemployed and receivers of social assistance.

## Pre-publication history

The pre-publication history for this paper can be accessed here:

http://www.biomedcentral.com/1471-2458/11/375/prepub
